# Quantitative proteomic approaches to studying redox post‐translational modifications in skeletal muscle

**DOI:** 10.1113/EP093434

**Published:** 2026-11-14

**Authors:** Sophie C. Broome, Elizabeth G. Reisman, Malcolm J. Jackson, John A. Hawley

**Affiliations:** ^1^ Center for Human Metabolism and Performance Mary MacKillop Institute for Health Research Australian Catholic University Melbourne Victoria Australia; ^2^ Australian Regenerative Medicine Institute (ARMI) Faculty of Medicine, Nursing and Health Sciences Monash University Victoria Australia; ^3^ Department of Musculoskeletal and Ageing Science Institute of Life Course and Medical Sciences University of Liverpool Liverpool UK; ^4^ Department of Sport and Exercise Sciences Manchester Metropolitan University Institute of Sport Manchester UK

**Keywords:** mass spectrometry, reactive oxygen species, redox proteomics, skeletal muscle

## Abstract

Reactive oxygen species are essential signalling molecules that regulate numerous aspects of skeletal muscle physiology. These effects are mediated through redox post‐translational modifications on protein cysteine thiols, which influence the structure and function of redox‐sensitive proteins. Mass spectrometry‐based redox proteomic approaches have greatly advanced our ability to detect and characterise cysteine redox modifications, revealing a broad network of redox‐sensitive proteins and pathways in skeletal muscle. Recent methodological developments enable quantification of the stoichiometry of reversible oxidative modifications at specific cysteine residues, providing critical insight into the extent and functional relevance of site‐specific redox regulation. Redox proteomic approaches are being employed to improve our understanding of the specific redox protein modifications underlying physiological and pathophysiological processes in skeletal muscle. This review summarises current proteomic strategies for quantifying redox post‐translational modifications and their application to studying redox signalling in skeletal muscle. Emerging experimental approaches that offer the potential to study the specific roles of site‐specific redox modifications in muscle physiology are also discussed. Collectively, these technologies present exciting opportunities to define the mechanistic roles of individual cysteine residues in muscle biology and help uncover new therapeutic avenues for conditions characterised by impaired redox homeostasis.

## Abstract (highlights)

- **What is the topic of this review?**
  The review explores quantitative proteomic approaches for identifying and characterising redox post‐translational modifications in skeletal muscle.
- **What advances does it highlight?**
  This review highlights recent advances in proteomic techniques to measure redox post‐translational modifications in biological samples and describes their application to studying redox signalling in skeletal muscle. It also highlights emerging methods for studying the roles of specific redox post‐translational modifications in muscle physiology, providing the opportunity to understand the mechanisms linking reactive oxygen species to muscle physiology and pathophysiology.

## 1 INTRODUCTION

Reactive oxygen species (ROS) and related reactive nitrogen and sulphur species (RNS/RSS) play a central role in skeletal muscle health and disease. Under conditions of oxidative distress, excessive ROS formation can cause irreversible damage to proteins, lipids and DNA, altering the structure and function of biomolecules (Kowalska et al., 2020; Lourenço Dos Santos et al., 2015; Panov & Dikalov, 2020). As a result, oxidative distress has been implicated in the pathogenesis of muscle atrophy and insulin resistance (Lourenço Dos Santos et al., 2015; Min et al., 2011; Souto P, 2015). Beyond causing oxidative damage, ROS, specifically hydrogen peroxide (H2O2), play an important role in regulating skeletal muscle physiology through reversible redox post‐translational modifications (PTMs) (Kramer et al., 2015), which provide a mechanism for cells to rapidly respond to changes in the cellular redox state. Redox PTMs influence diverse aspects of protein function, including catalytic or ligand binding activities, protein–protein interactions and protein stability, while also regulating the formation of other PTMs associated with physiological signalling (Su et al., 2019).

Many metabolic and myofibrillar proteins in skeletal muscle contain redox‐sensitive cysteine residues (Kramer et al., 2018; McDonagh et al., 2014), implicating redox PTMs in the regulation of muscle metabolism and force production. For example, peroxynitrite‐induced oxidative modification of Cys278 of mitochondrial creatine kinase inactivates the enzyme with downstream impacts on cellular energy homeostasis and calcium handling (Stachowiak et al., 1998). The skeletal muscle calcium release channel ryanodine receptor (RyR1) also contains many free thiols whose oxidation or nitrosylation influences channel function. At physiological $P_{O_{2}}$, nanomolar nitric oxide activates RyR1 by *S*‐nitrosylation of Cys3635 (Eu et al., 2000; Sun et al., 2001), linking redox signalling to muscle contractility. Furthermore, mechanical unfolding of titin immunoglobulin (Ig) domains exposes buried cysteine residues, which then can be *S*‐glutathionylated (Alegre‐Cebollada et al., 2014). *S*‐glutathionylation of these cryptic cysteines decreases the mechanical stability of the parent Ig domain as well as its ability to fold, leading to reduced titin stiffness and increased muscle elasticity. Changes in the cellular redox state also enable skeletal muscle to adapt to diverse stimuli, including contractile activity, the prevailing loading conditions, substrate availability and environmental factors (e.g., hypoxia, heat and cold stress). Muscle contraction results in transient increases in the cytosolic H2O2 content of muscle fibres, which, in part, mediate exercise‐induced mitochondrial biogenesis, angiogenesis and upregulation of endogenous antioxidant enzymes through the activation of redox‐sensitive signalling pathways (Henriquez‐Olguin et al., 2023; Margaritelis et al., 2020).

Despite the importance of redox regulation in skeletal muscle physiology and pathophysiology, our understanding of the specific redox PTMs that explain the molecular basis for these processes is limited, in part because methods for studying redox PTMs have typically not provided absolute stoichiometric quantitation and/or cover a small proportion of the redox proteome. Furthermore, experimental approaches to investigate causal links between redox PTMs and biological outcomes lack precision and can disrupt non‐redox cysteine roles. However, in recent years, advances in redox proteomic approaches have enabled the quantification of reversible oxidative modifications on specific protein cysteine residues in a range of tissues, including skeletal muscle, with increasing coverage of the cysteine proteome. In this review, we discuss proteomic strategies to quantify redox PTMs and their application to study redox signalling in skeletal muscle. We also highlight emerging methodologies that selectively induce or block cysteine redox modifications and offer the potential to study the role of site‐specific modifications in muscle physiology.

## 2 REGULATION OF THE SKELETAL MUSCLE REDOX STATE AND REDOX SIGNALLING MECHANISMS

The skeletal muscle redox state is determined by the balance between ROS production and antioxidant defence systems. Superoxide, the primary ROS generated in muscle, is produced by multiple sources including mitochondria, the endoplasmic reticulum, peroxisomes and enzymes such as NADPH oxidases (NOX). Superoxide is rapidly converted to H2O2, which is membrane‐permeant and functions as a key signalling molecule due to its selective reactivity with cysteine thiols. H2O2 is subsequently converted to water, or water and oxygen, by compartmentalised antioxidant enzymes including catalase (CAT), peroxiredoxins (PRDX) and glutathione peroxidases.

The mitochondrial electron transport chain (ETC) is a major source of superoxide (Murphy, 2009), and mitochondrial redox signalling plays important roles in a wide range of biological processes (Collins et al., 2012). Furthermore, disruptions to mitochondrial redox homeostasis have been linked to muscle atrophy (Min et al., 2011) and insulin resistance (Fiorenza et al., 2024). Although muscle contraction alters the redox state of mitochondrial proteins (Pugh et al., 2021; Stretton et al., 2020), mitochondria do not appear to be the primary source of increased ROS during contraction (Henríquez‐Olguin et al., 2019). NOX2 is a key source of contraction‐induced H2O2 (Henríquez‐Olguin et al., 2019), regulating glucose uptake (Henríquez‐Olguin et al., 2019) and mitochondrial adaptations to exercise training (Henríquez‐Olguín et al., 2019). However, NOX2‐derived ROS have also been implicated in muscle atrophy (Lawler et al., 2021) and insulin resistance (Souto, 2015), underscoring the divergent effects of ROS generated from the same source under distinct physiological and pathophysiological conditions. H2O2 derived from NOX4, a constitutively active isoform localised to the sarcoplasmic reticulum (Sakellariou et al., 2013), also contributes to exercise‐induced redox signalling and promotes adaptive responses that improve muscle function, maintain redox balance and prevent the development of insulin resistance (Xirouchaki et al., 2021).

H2O2 exerts its effects through reversible oxidation of cysteine residues on target proteins. Redox‐sensitive cysteine thiols can undergo a wide range of reversible and irreversible redox modifications, including sulfenylation, nitrosylation, glutathionylation and disulfide formation (Figure 1). The disulfide proteome is made up of structural disulfides and reversible disulfides (Sun et al., 2017). Unlike structural disulfides, reversible disulfides can have important functional roles and may serve as redox switches. Sulfenylated thiols can undergo further oxidation to form stable sulfinic or sulfonic acids, which can be used as markers of oxidative damage. These modifications alter protein activity, structure and interactions and regulate diverse cellular processes, including intracellular signalling, metabolism and gene expression (Lennicke & Cochemé, 2021). The reactivity of redox regulated proteins to H2O2 is generally considered to be significantly lower than that of H2O2‐scavenging peroxidases. Thus, several scenarios could explain the potential mechanism by which H2O2 oxidises low‐reactivity target proteins. Transient inactivation of PRDXs by reversible hyperoxidation or phosphorylation may result in increases in intracellular H2O2 that allow direct oxidation of protein thiols with low H2O2 reactivity (known as the ‘floodgate’ theory). The other potential mechanism whereby H2O2 may oxidise low‐reactivity target proteins is known as a ‘redox relay’, which involves PRDXs transferring oxidising equivalents to less reactive thiol proteins in redox signalling pathways. Redox relays have been identified with the transcription factors apoptosis signal‐regulating kinase 1 and signal transducer and activator of transcription 3 (STAT3) with PRDX1 and PRDX2, respectively (Jarvis et al., 2012; Sobotta et al., 2015).

**FIGURE 1:**
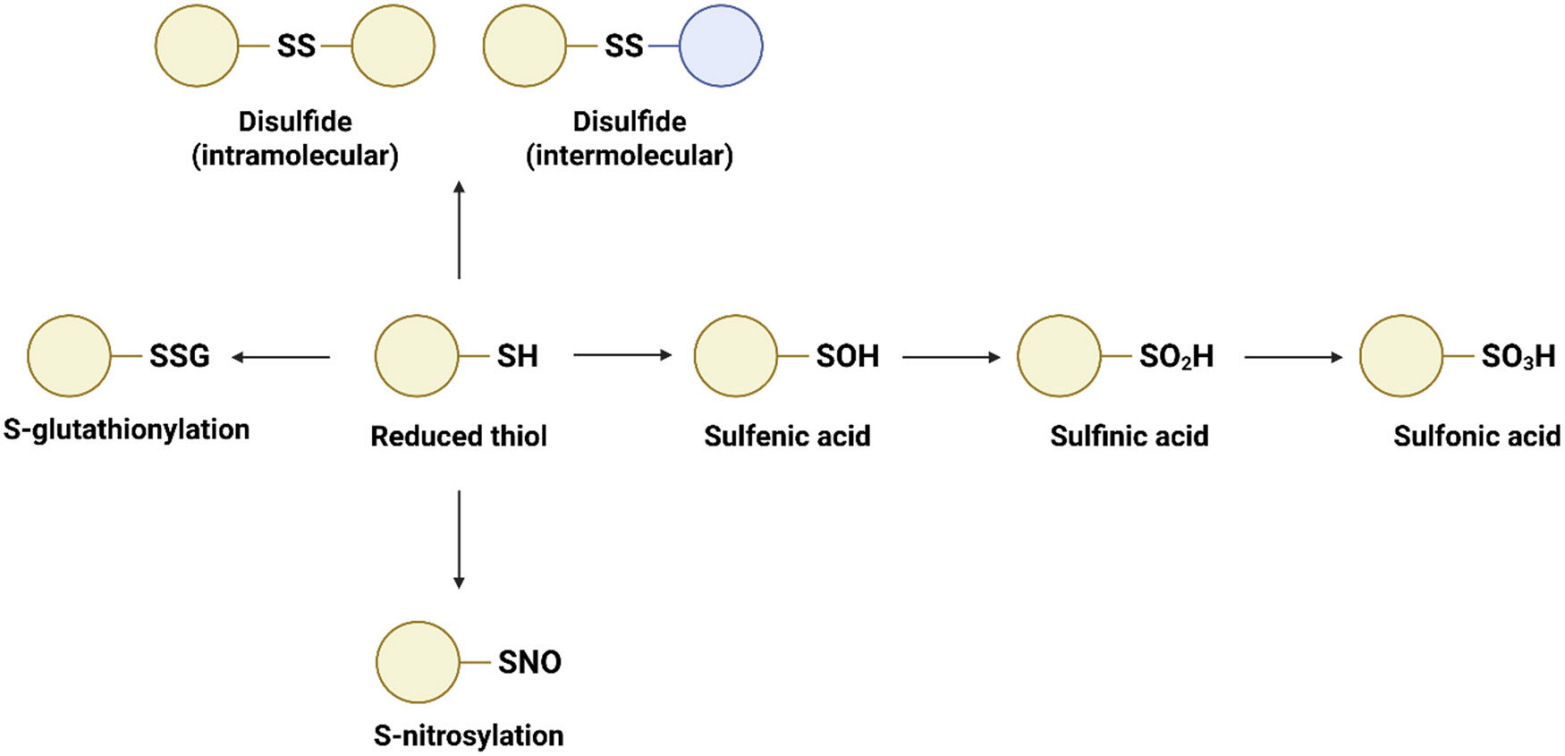
Oxidative modifications of protein cysteine thiols. Reduced thiols can be subjected to multiple types of oxidative modifications. Disulfides can be formed intramolecularly within the same protein or intermolecularly through exchange with a sulfur group on another protein or molecule. Sulfinylation and sulfonylation occur as subsequent, irreversible oxidative modifications of cysteine thiols that are sulfenylated. Apart from sulfinylation and sulfonylation, all modifications illustrated here are considered reversible. Figure created with BioRender.

## 3 QUANTITATIVE PROTEOMIC APPROACHES FOR MEASURING REVERSIBLE REDOX PTMS: APPLICATION TO SKELETAL MUSCLE PHYSIOLOGY

While redox regulation plays a fundamental role in skeletal muscle physiology and pathophysiology, the underlying protein modifications remain poorly defined. Efforts to characterise the skeletal muscle redox proteome have been constrained because redox proteomics methods have typically not provided absolute stoichiometric quantitation of cysteine oxidation, preventing determination of the percentage occupancy of oxidation at specific cysteine sites. PTM site stoichiometry, also referred to as PTM site occupancy or fractional PTM occupancy, indicates the fraction of a protein that is modified with a given PTM at a specific site (Prus et al., 2019) (Figure 2). Because many redox‐sensitive cysteine residues function as allosteric or structural switches (Barford, 2004), small shifts in oxidation stoichiometry can have large effects on muscle function. Therefore, accurately quantifying the extent of reversible cysteine oxidation is essential to interpret how redox signals are translated into physiological responses and to identify key functional sites through which ROS and related species exert their physiological roles. Current stoichiometric approaches generally provide limited coverage of the cysteine proteome, identifying only a fraction of cysteine‐containing peptides or proteins, which limits identification of key functional cysteine redox switches. Furthermore, few redox proteomic approaches allow for the determination of specific reversible redox modifications, such as *S*‐glutathionylation and *S*‐nitrosylation. These technical challenges have limited our ability to quantitatively interrogate how protein redox modifications regulate muscle physiology. However, advances in mass spectrometry (MS)‐based redox proteomics have made it possible to quantify redox PTMs on protein cysteine thiols with increasing proteome coverage. A comparison of proteomic approaches that have been used to quantify reversible cysteine redox modifications in skeletal muscle is provided in Table 1.

**FIGURE 2:**
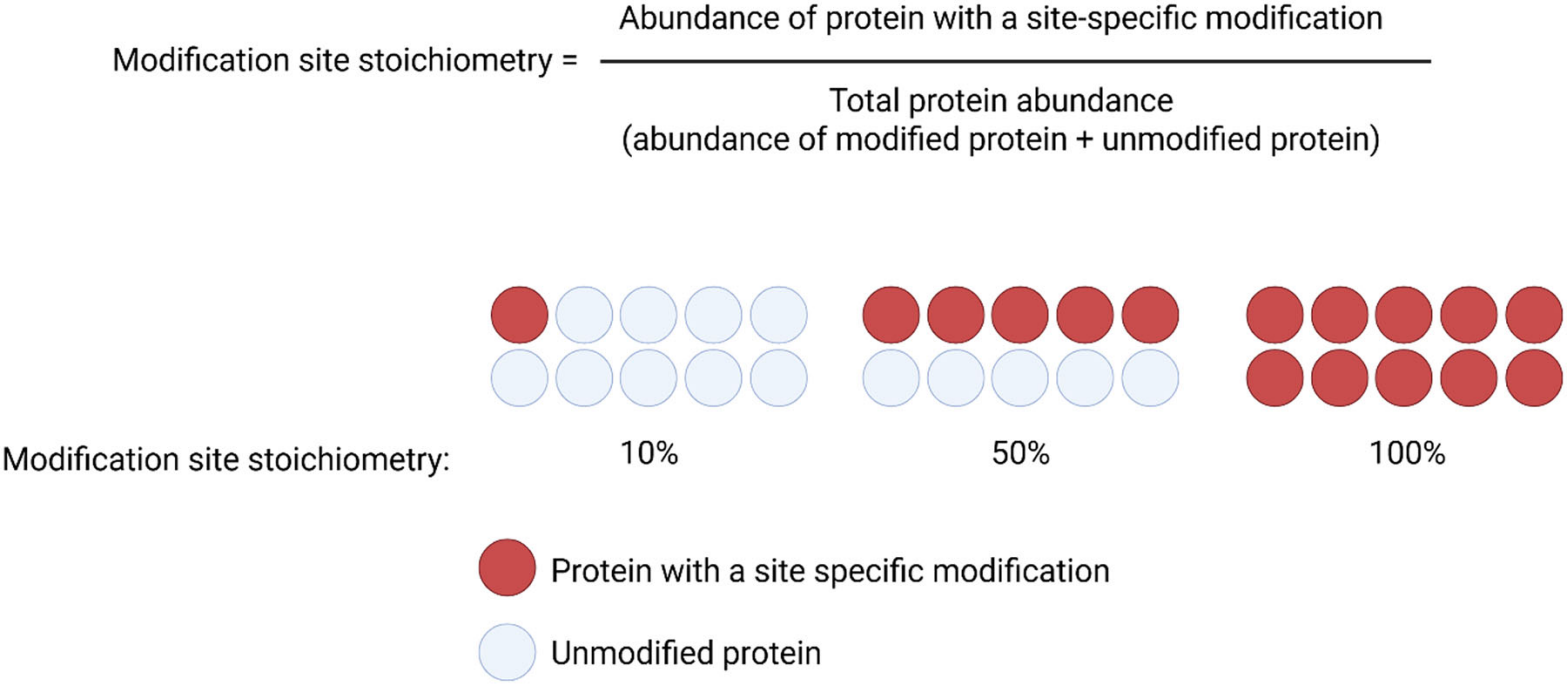
Protein modification site stoichiometry. The concept of post‐translational modification (PTM) site stoichiometry is illustrated using three examples with different PTM site stoichiometry. Unmodified and PTM‐containing proteins are marked as indicated. Figure created with BioRender.

**TABLE 1:** Comparison of proteomic approaches for quantifying reversible cysteine redox modifications.

Workflow	Labelling/enrichment strategy	Advantages	Limitations	Examples of application in skeletal muscle
Differential cysteine labelling (NEM)	d0‐NEM labels reduced cysteine thiols; reversibly oxidised cysteine thiols are subsequently reduced; d5‐NEM labels cysteine thiols that were reversibly oxidised	Enables site‐specific measurement of relative cysteine oxidation	Generally low coverage; poor sensitivity for low‐abundance proteins; not readily multiplexed; cannot identify the specific reversible redox modification	Ageing and acute exercise‐induced changes in human and mouse skeletal muscle cysteine oxidation (McDonagh et al. 2014; Pugh et al. 2021; Smith et al. 2018)
Resin‐assisted capture	Free cysteine thiols are initially blocked with NEM; oxidised cysteine thiols are reduced; the resulting free cysteine thiols are captured by thiol‐affinity resin	Enriches modified proteins/peptides, reducing sample complexity and improving coverage; specific reducing reagents can be used to target modifications such as *S*‐glutathionylation or *S*‐nitrosylation; compatible with multiplexed TMT quantification	Incomplete blocking and non‐specific oxidation during sample handling can affect accuracy	*S*‐glutathionylation following muscle contraction; age‐related *S*‐glutathionylation; deep profiling of cysteine oxidation in muscular dystrophy and older individuals (Campbell et al. 2019; Day et al. 2022, 2024; Kramer et al. 2018)
Cysteine‐reactive phosphate tags	Cysteine‐reactive labelling reagent is used to label cysteine‐containing peptides; the phosphate‐containing tag enables IMAC‐based enrichment	Strong enrichment of cysteine‐containing peptides; improves recovery of low‐abundance peptides; IMAC‐based enrichment can substantially increase redox proteome coverage; compatible with TMT, workflow could be adapted to enrich thiols that have been selectively reduced to probe for a specific redox modification	Non‐specific oxidation during sample handling can affect accuracy	Age‐associated cysteine oxidation across tissues, including skeletal muscle; insulin‐stimulated changes in skeletal muscle cysteine oxidation (Henríquez‐Olguin et al. 2025; Xiao et al. 2020)

Abbreviations: IMAC, immobilised metal affinity chromatography; NEM, *N*‐ethylmaleimide; TMT, tandem mass tag.

Label‐free approaches and isotopic and isobaric labelling make redox proteomics quantitative. Label‐free proteomics measures protein abundance directly from the MS signal intensity or spectral counts across separate liquid chromatography (LC)‐MS runs. Quantitative proteomic analyses are commonly performed using data‐dependent acquisition (DDA), in which precursor ions are selected for fragmentation based on intensity and identified using spectral libraries. However, data‐independent acquisition (DIA) has become increasingly important due to its improved reproducibility and depth, particularly in enriched samples such as those generated by redox proteomic workflows (Bruderer et al., 2017). In isotopic labelling, stable isotopes are incorporated into proteins or peptides, creating a defined mass shift that allows relative quantification based on signal intensity. Isobaric labelling methods use chemical tags, such as tandem mass tags (TMT) (Thompson et al., 2003) or isobaric tags for relative and absolute quantification (iTRAQ) (Ross et al., 2004), which release distinct reporter ions upon fragmentation, enabling multiplexed quantification of protein abundances in several samples in a single run, increasing throughput and reducing LC–tandem mass spectrometry (MS/MS) run variability. To date, most studies that have used isobaric labelling to quantify cysteine oxidation in skeletal muscle have employed TMT labelling. In this approach, each peptide sample is chemically labelled with TMT consisting of three parts: a reporter group that differs in isotopic composition, a balance group that equalizes the total mass across tags, and a reactive group that covalently binds to peptide N‐termini or lysine residues. After labelling, the samples are combined and analysed by LC‐MS/MS. Because the labelled peptides are isobaric, they appear as a single precursor ion, ensuring identical chromatographic and ionization behaviour. During fragmentation, each tag releases its unique reporter ion, and the relative intensities of these reporter ions reflect the abundance of the peptide in each original sample. This approach provides precise, high‐throughput quantification across multiple biological conditions.

### 3.1 Differential cysteine labelling

Differential labelling techniques involve first blocking free cysteine thiols with an alkylating reagent followed by reduction of reversibly oxidised cysteines and a second alkylation step using a chemically distinct or isotopically labelled reagent. This two‐step labelling process allows reduced and oxidised cysteines to be distinguished by MS based on their differential mass tags, enabling site‐specific quantification of redox‐sensitive cysteine residues (Figure 3). Isotope‐coded affinity tag (ICAT) reagent is a chemical probe that labels reduced thiols due to its iodoacetamide moiety, which rapidly and irreversibly reacts with free thiols (Leichert et al., 2008). Once free thiols are labelled with the light variant of the ICAT reagent, reversibly oxidised thiols in the same sample are reduced and labelled with the heavy ICAT reagent. Following digestion and purification of the labelled peptides by their biotin tags, the enriched sample is analysed using MS/MS, where the reduced and oxidised peptides can be resolved based on their mass shift of 9 Da, which is visible on the spectra. Finally, the stoichiometry of oxidised versus reduced proteins is determined by the ratio of heavy versus light peptide signal intensities.

**FIGURE 3:**
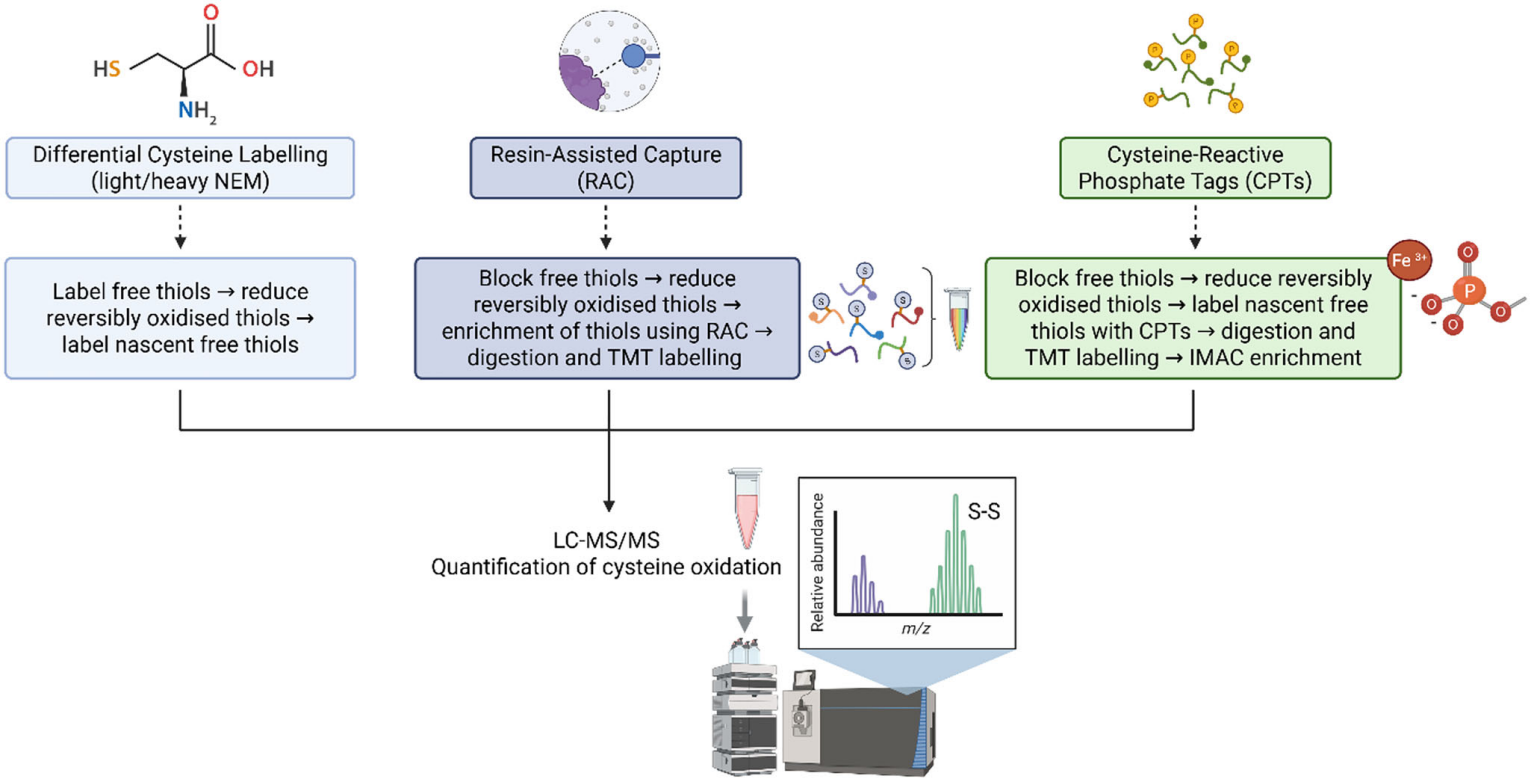
Quantitative redox proteomic workflows to study protein cysteine oxidation in skeletal muscle. The differential cysteine labelling using NEM approach involves labelling free thiols using *N*‐ethylmaleimide (d0‐NEM) before reversibly oxidised cysteines are reduced and the newly formed free thiols are labelled with heavy NEM (d5‐NEM). Relative quantification of cysteine containing peptides identified as reduced (d0‐NEM labelled) and reversibly oxidised (d5‐NEM labelled) is then performed using the intensity of their precursor ions. In the cysteine‐reactive phosphate tag (CPT) and resin assisted capture (RAC) methods, total thiols (i.e., both oxidised and reduced protein thiols) are measured in a separate sample by omitting the initial step to block free thiols for stoichiometric measurements. Measurements are based on reporter ion intensities. In the RAC workflow, free thiols are alkylated, and then oxidised thiols are totally or selectively reduced (if probing for a specific modification). RAC of nascent free thiols using a thiopropyl sepharose resin is followed by on‐resin digestion and labelling with tandem mass tags (TMT). Finally, peptides are eluted off the resin prior to analysis by mass spectrometry (MS). The oxidation occupancy is determined by taking the ratio of reporter ion intensities in the oxidised and total thiol channels to evaluate the modification stoichiometry. In the CPT workflow, free thiols are alkylated and oxidised thiols are totally or selectively reduced. Endogenous phosphorylation is removed to avoid the recovery of phosphorylated peptides during immobilised metal affinity chromatography (IMAC). Nascent free thiols are labelled with CPTs before the proteins are digested and the peptides are labelled with TMT. The CPTs allow the peptides to be enriched by IMAC and are eluted off the column prior to analysis by MS. The oxidation occupancy is determined by taking the ratio of reporter ion intensities in the oxidised and total thiol channels to evaluate the modification stoichiometry. Figure created with BioRender.

Another differential labelling approach involves alkylating free thiols using *N*‐ethylmaleimide (d0‐NEM) before reducing reversibly oxidised cysteines and labelling the newly formed free thiols with heavy NEM (d5‐NEM) (McDonagh et al., 2014). Relative quantification of cysteine containing peptides identified as reduced (d0‐NEM labelled) and reversibly oxidised (d5‐NEM labelled) is then performed using the intensity of their precursor ions. Differential cysteine labelling has been combined with label‐free proteomics to quantify age‐related changes in protein abundance and the reversible oxidation state of redox‐sensitive cysteines in mouse skeletal muscle (McDonagh et al., 2014; Smith et al., 2018). Many mitochondrial and myofibrillar proteins were among the proteins with redox sensitive thiols detected in these studies. While there was greater oxidation of some cysteines in aged muscle, others shifted toward to a more reduced state, highlighting that ageing is associated with remodelling of the redox proteome rather than a global increase in oxidation. Notably, changes in the redox proteome appear to be well preserved across gastrocnemius, quadriceps and soleus muscles in rodents with 23 cysteine residues showing altered redox states in all three despite their different fibre type compositions and susceptibility to ageing. The total number of proteins containing redox‐sensitive cysteines decreased in aged muscle, suggesting a loss of redox signalling flexibility. For example, in the gastrocnemius, proteins that were identified as containing redox‐sensitive cysteine residues in muscle from adult mice only included proteins involved in glucose metabolism, such as phosphofructokinase, glucose 6‐phosphate isomerase, glycogen phosphorylase, phosphoglycerate mutase 1 and phosphoglucomutase 2 (McDonagh et al., 2014). Cysteine residues regulate the activity of many of these enzymes and the loss of redox sensitivity at these sites in aged muscle may reflect reduced flexibility in metabolic redox regulation with age. This loss of flexibility in redox regulation is mirrored by a loss of skeletal muscle metabolic flexibility with ageing in which the capacity to switch from carbohydrate‐ and fat‐based fuels in response to the prevailing hormonal milieu is diminished (Nunn et al., 2009).

Differential alkylation using NEM has also been employed to investigate the effects of ageing and exercise on the human skeletal muscle redox proteome (Pugh et al., 2021). Compared with mice, humans demonstrate fewer age‐related shifts in the redox state of individual cysteines. All significant differences observed at rest showed a decrease in relative cysteine oxidation in aged muscle (i.e., cysteines were in a more reduced state). Ageing was associated with decreased oxidation of seven cysteines from five different proteins, including glycogen phosphorylase, phosphoglycerate mutase 2, PRDX6, ubiquitin‐conjugating enzyme E2 N and Rab GDP dissociation inhibitor. The shift in specific cysteine residues toward a more reduced state appears counterintuitive as previous studies have reported increased irreversible protein oxidation in aged muscle (Lourenço Dos Santos et al., 2015). However, such an observation is consistent with studies in mouse muscle showing age‐related shifts to a more reduced state in cytosolic proteins, such as PRDX2 (Stretton et al., 2020). The mechanisms underpinning this greater reduction of cysteine residues in aged muscle are unclear but may involve adaptive upregulation of cytoprotective antioxidant proteins in response to elevated mitochondrial oxidative stress with age (Jackson, 2020).

While there appear to be few differences in protein cysteine oxidation between young and old individuals at rest, there is a greater disparity in the response to an acute bout of high‐intensity interval exercise. In muscle from both young and older individuals, four cysteines from three mitochondrial proteins (dihydrolipoamide dehydrogenase, cytochrome *b*–*c*_1_ complex subunit 1 (UQCRH) and PRDX3) exhibited increased oxidation in response to exercise, while one cysteine showed a reduction. A further 23 cysteines from 13 proteins exhibited exercise‐induced oxidation in muscle from older individuals only, including proteins involved in glycolysis (aldolase A, beta‐enolase (ENO3), glyceraldehyde‐3‐phosphate dehydrogenase (GAPDH), and triosephosphate isomerase 1), ROS detoxification (CAT, PRDX6 and superoxide dismutase 1) and creatine kinase activity (creatine kinase M‐type and S‐type). Nine of these proteins are cytosolic, while the rest are mitochondrial or located within the peroxisome, indicating exercise results in changes in the redox state of proteins localised to several subcellular compartments in aged muscle. Taken together, these findings suggest that redox homeostasis is largely preserved during exercise in young muscle, whereas there appears to be a disruption of the redox proteome during exercise in aged muscle.

Differential cysteine labelling using NEM has provided insight into age‐ and exercise‐related changes in protein cysteine oxidation in skeletal muscle. However, compared to techniques that purify/enrich modified cysteine containing peptides, this technique has a relatively poor sensitivity for the detection of low‐abundance proteins as labelled peptides compete with extremely abundant sarcomeric proteins, reducing the dynamic range. Indeed, this approach only identified 75 redox cysteine peptides on 45 proteins in adult mouse quadriceps (Smith et al., 2018), and in the human muscle biopsy study 154 redox cysteine peptides in 57 proteins were reported (Pugh et al., 2021). Increasing the sensitivity of detection by sample fractionation or data independent approaches would increase the accuracy of both detection and quantification of redox specific changes. Differential cysteine labelling using NEM is not suitable for multiplexing, which limits analysis to only two samples at a time, decreasing throughput and potentially increasing LC‐MS/MS run variability. Furthermore, precursor‐level quantification (MS1) used in the NEM differential alkylation workflow has lower precision than TMT/MS2‐based quantification, particularly for detecting small redox shifts. These differential alkylation approaches do not allow for determination of specific reversible redox modifications, limiting the capacity to elucidate the specific protein modifications underlying muscle physiology.

### 3.2 Resin‐assisted capture

As discussed, maximising cysteine proteome coverage is a major technical challenge in the field of redox proteomics. Resin‐assisted capture (RAC) enables the enrichment of oxidatively modified proteins, increasing the coverage of downstream proteomic analysis (Gaffrey et al., 2021). In this approach, protein free thiols are initially blocked with NEM before reversibly oxidised thiols are reduced and proteins with nascent free thiols are enriched using a thiol‐affinity resin (Figure 3). RAC has the versatility to profile specific modifications such as glutathionylation or nitrosylation by using specific reagents to selectively reduce them (Kramer et al., 2018). The captured proteins are digested on‐resin with trypsin, and the peptides are labelled on‐resin with TMT. Direct capture of thiol‐containing proteins reduces sample complexity and non‐specific background, improving digestion efficiency and TMT incorporation onto the remaining cysteine containing peptides. When RAC is combined with TMT labelling (RAC‐TMT), changes in the redox state of oxidatively modified proteins can be evaluated quantitatively.

The RAC‐TMT workflow has been employed to investigate the effect of contractile activity on protein *S*‐glutathionylation in mouse muscle (Kramer et al., 2018). Over 2200 sites of *S*‐glutathionylation modifications were detected, of which 1290 were significantly increased after contractions. Regulatory cysteines on metabolic proteins were *S*‐glutathionylated, including mitochondrial ETC complexes I, II and V (ATP synthase), GAPDH, malate dehydrogenase 1 and aconitase 2. *S*‐glutathionylation of these proteins is known to regulate their activity. For example, contraction resulted in *S*‐glutathionylation of Cys150 in the key glycolytic enzyme GAPDH, which has been shown to inactivate the enzyme and alter its tertiary structure (Barinova et al., 2017). Furthermore, ATP synthase, which is responsible for utilising the proton‐motive force for ATP production, was *S*‐glutathionylated at Cys244 and Cys294 of the α‐subunit following contractions. *S*‐glutathionylation of Cys294, although initially inhibitory, provides protection from the irreversible modification of sulfenic to sulfinic to sulfonic acid formation (Mailloux & Willmore, 2014). Muscle contractions also resulted in *S*‐glutathionylation of excitation–contraction coupling proteins RyR1 and sarco/endoplasmic reticulum calcium ATPase 1 (SERCA1), and myofibrillar proteins titin and troponin I2. Oxidative modification of excitation–contraction coupling and myofibrillar proteins can have an immediate effect on contraction and may be responsible for some of the changes in contraction kinetics observed during exercise‐induced fatigue. For example, *S*‐glutathionylation of SERCA1 at Cys674 can disrupt calcium reuptake into the sarcoplasmic reticulum and impact muscle relaxation (Adachi et al., 2004). The largest fold changes in the *S*‐glutathionylated proteome after contractions occurred on signalling proteins such as 14‐3‐3 protein gamma (YWHAG) and mitogen‐activated protein kinase 4, as well as proteins like SERCA and NADH:ubiquinone oxidoreductase subunit V2 of mitochondrial complex I (NDUFV2). Taken together, these findings highlight the important role of redox regulation in controlling muscle physiology, metabolism and the adaptive response to exercise.

Using the RAC‐TMT approach, Campbell and colleagues demonstrated that ageing is also associated with increased *S*‐glutathionylation of protein cysteine thiols in mouse skeletal muscle (Campbell et al., 2019). Among the biological processes affected, the TCA cycle showed the highest proportion of proteins with significantly altered *S*‐glutathionylation, followed by pathways involved in sarcomeric structure and assembly. Proteins mediating interactions between the intracellular and extracellular environments were especially vulnerable to age‐related shifts in redox status. Specifically, proteins associated with focal adhesion, the structural link between the plasma membrane and the extracellular matrix, as well as components of the myelin sheath in motor neurons, were highly modified in aged muscle. Focal adhesion molecules play a role in linking the sarcolemma to the extracellular matrix, which is critical for formation and stabilization of the neuromuscular junction (Li et al., 2018). This is of particular interest given the growing recognition of the importance of loss of motor units and disruption of the neuromuscular junction in sarcopenia (Rudolf et al., 2014). Mitochondrial proteins were also highly sensitive to age‐related increases in *S*‐glutathionylation with almost one‐third of all cysteine sites displaying significant alterations in *S*‐glutathionylation located in or associated with the mitochondria. Notably, treatment of aged mice with the mitochondria‐targeted compound SS‐31 (elamipretide) restored redox homeostasis in skeletal muscle, and the profile of *S*‐glutathionylation on skeletal muscle proteins in aged mice treated with SS‐31 closely resembled that of young mice. Reversal of these modifications by SS‐31 treatment strongly supports an important role for mitochondrial redox regulation in age‐related protein modifications.

To enhance coverage of the redox proteome, the RAC‐TMT workflow has been modified to incorporate a microscale fractionation scheme to further reduce sample complexity and improve the detection of redox‐sensitive peptides (Day et al., 2022). This workflow has been applied to investigate changes in protein cysteine oxidation in a mouse model of muscular dystrophy (Day et al., 2022). In‐depth coverage of the redox proteome was achieved with >18,000 Cys sites from 5608 proteins in muscle being quantified. Notably, this level of coverage was enabled by the incorporation of offline fractionation strategies, which are increasingly essential for achieving deep and reproducible coverage in redox proteomics and may also be required to maximise site depth in cysteine‐reactive phosphate tag‐based workflows. Compared to the control group, *mdx* mice exhibited a marked increase in cysteine oxidation, where a ∼2% shift in the median oxidation occupancy was observed. Marked changes in cysteine oxidation were seen in proteins associated with coagulation in *mdx* mice. Specifically, cysteines on proteins involved in the formation of blood clots, including fibrinogen β/γ chain, von Willebrand factor, plasminogen, prothrombin and thrombomodulin, displayed greater oxidation in *mdx* mice. Abnormalities or disorders in blood coagulation are side effects of Duchenne muscular dystrophy (Saito et al., 2001). While the underlying molecular mechanisms linking Duchenne muscular dystrophy and blood coagulation abnormalities are unclear, these findings suggest that oxidative stress may be involved. Increased cysteine oxidation was also observed in proteins involved in phagosome degradation by lysosomes, which may contribute to autophagic dysfunction in *mdx* mice. Autophagy is vital for homeostasis of muscle tissue and skeletal muscle may succumb to degeneration when autophagy is impaired.

The RAC‐TMT workflow has been employed to investigate associations between skeletal muscle protein cysteine oxidation and physical performance in older individuals. Day et al. (2024) quantified the oxidation of 15,049 cysteine sites, covering 4354 proteins in skeletal muscle. Higher levels of cysteine oxidation on muscle contractile proteins were generally negatively associated with measures of muscle function, physical performance and fitness. For example, higher oxidation of cysteine sites in nebulin was associated with lower peak oxygen uptake (${\dot{V}}_{O_{2}\mathrm{peak}}$). Nebulin regulates the length of the sarcomeric thin filament by acting as a molecular ruler to establish a minimal length for filamentous actin (Labeit et al., 1991). Thus, oxidation of cysteines on nebulin may disrupt these essential interactions, preventing the organisation of actomyosin filaments with downstream implications for muscle contraction. Greater oxidation of cysteine sites in myomesin‐1, myomesin‐2 and nebulin was associated with slower walking speed, and higher oxidation of cysteine sites in myomesin‐2 and α‐actinin‐2 was associated with lower leg power and strength. Of note, oxidation of α‐actinin‐3 was positively associated with peak isotonic leg power. α‐Actinin proteins bind and crosslink overlapping F‐actin filaments at the Z‐line in the sarcomere (Burgoyne, Morris et al., 2015), making them important proteins for muscle integrity and quality. Therefore, oxidation in the different functional domains of α‐actinin‐3 may regulate its function in generating maximum contractile power. Despite this observation, oxidation of cysteines on muscle proteins was generally associated with impaired muscle function and physical performance in older individuals. These findings provide a potential mechanism underlying the links between oxidative stress and declines in muscle function and mobility during ageing.

### 3.3 Cysteine‐reactive phosphate tags

While redox proteomics typically identifies up to several thousand cysteine sites, phosphoproteomic approaches achieve greater coverage, highlighting significant differences in the capacity of these approaches to recover proteins of interest. To improve the enrichment of cysteine‐containing proteins from tissue extracts, cysteine‐reactive reagents have been chemically modified to incorporate tags that facilitate their selective capture (Figure 3). Notably, the cysteine‐reactive compound iodoacetamide has been modified to carry a phosphate group, allowing labelled peptides to be purified using immobilised metal affinity chromatography (IMAC) – a technique commonly used for phosphopeptide enrichment. IMAC‐based enrichment enhances coverage because the phosphate group on the modified cysteine provides a strong, specific affinity handle for metal ions such as Fe^3^^+^ or Ti^4^^+^, enabling efficient and selective pull‐down of labelled peptides. The use of cysteine‐reactive phosphate tags (CPTs) improves recovery of low‐abundance cysteine‐containing peptides, thereby increasing coverage of the redox proteome. Furthermore, while this technique has only been used to detect total oxidised cysteines, it could also be used to enrich thiols that have been selectively reduced to probe for a specific redox modification.

Using CPTs combined with TMT multiplexing, Xiao and colleagues measured protein cysteine oxidation across 10 mouse tissues to investigate age‐associated redox changes (Xiao et al., 2020). They quantified the oxidation of ∼34,000 unique cysteine sites on ∼9400 proteins across all tissues, including ∼4500 cysteine sites in skeletal muscle. Similar to previous studies employing differential cysteine labelling techniques (McDonagh et al., 2014; Smith et al., 2018), their data revealed that cysteines involved in tissue‐specific redox regulation were lost with age. Moreover, aged tissues displayed distinct, tissue‐specific remodelling of the redox signalling landscape. In skeletal muscle, ageing was associated with greater oxidation of cysteines on protein drivers of muscular dystrophies, including Cys774 on collagen α‐3(VI) chain (COL6A3), Cys630 and Cys1981 on dysferlin, Cys321 on myotilin and several sites in titin – Cys5368, Cys6920, Cys7512, Cys13231, Cys21834, Cys20367 and Cys21689. These results provide a molecular basis for the proposed links between redox dysregulation and specific functional age‐related declines in skeletal muscle.

CPTs have also been employed to investigate the role of redox signalling in insulin‐stimulated glucose uptake in skeletal muscle (Henríquez‐Olguín et al., 2025). Despite inconsistent evidence, there is a longstanding belief that insulin signalling increases H_2_O_2_ production, leading to reversible oxidation of cysteine thiols and stimulation of intracellular phospho‐signalling downstream of the insulin receptor. However, the results of a recent study that utilised advanced redox tools to study the effects insulin stimulation on H_2_O_2_ levels and protein cysteine oxidation in mouse skeletal muscle challenged this notion. Using genetically encoded H_2_O_2_ sensors and non‐reducing immunoblotting, Henríquez‐Olguín et al. (2025) observed no increase in skeletal muscle subcellular H_2_O_2_ levels following insulin stimulation. Furthermore, stoichiometric cysteine proteome analyses using CPTs combined with TMT multiplexing revealed a selective pro‐reductive shift in cysteine modifications affecting insulin transduction‐related proteins. Specifically, Cys179 on glycogen synthase kinase‐3 β, a classical insulin‐inhibited kinase, was selectively reduced by insulin, in addition to other cysteines on known insulin‐action‐related proteins such as Cys416 on Ras and Rab interactor 2 and Cys450 on supervillin. The selective reduction of these proteins correlated with the glucose‐lowering effect of insulin. Together, these findings suggest that an insulin‐stimulated pro‐reductive shift modulates specific aspects of insulin signal transduction.

### 3.4 Challenges with redox proteomic approaches

A major challenge in redox proteomics is the accurate capture of reversible cysteine PTMs, which are highly sensitive to experimental handling. Cysteines can undergo artificial oxidation during sample collection, storage and processing, leading to distortion of the in vivo redox state, which limits technical validity and reproducibility. Preventing these artefacts requires stringent control during the pre‐analytical phase, including blocking of native free thiols with an alkylating agent such as NEM to preserve redox status. Following the selective reduction of reversibly oxidised thiols, it is still possible that the nascent free thiols can be re‐oxidised during subsequent sample processing steps to form inter‐ or intra‐molecular disulfides. This is an ever‐present issue for the methods described above as they all employ selective reduction of oxidised thiols. In the case of RAC, a small concentration of dithiothreitol is retained in the buffer during the capture step to prevent such issues and to ensure high recovery of formerly oxidised peptides. Despite these precautions, incomplete thiol blocking and non‐specific oxidation remain significant technical limitations. These challenges highlight the need for carefully optimised methodologies to accurately and reliably quantify redox PTMs and enable meaningful biological interpretation.

Another limitation of redox proteomic approaches is their relative inaccessibility to most researchers. Microplate assays that can measure protein thiol redox state have been developed and present an alternative method for studying redox signalling in skeletal muscle. The antibody‐linked oxi‐state assay (ALISA) is an enzyme‐linked immunosorbent assay (ELISA)‐based method designed to measure a target‐specific protein thiol redox state (Noble et al., 2021). In this approach, reduced thiols are alkylated to preserve the native redox status. Reversibly oxidised thiols can then be selectively reduced and labelled with fluorescent maleimide (F‐MAL). Samples are then incubated with a capture antibody, and the capture antibody–target complex is labelled with an amine‐reactive fluorescent *N*‐hydroxysuccinimide ester (F‐NHS). The target is then selectively eluted, and target specific F‐MAL and F‐NHS fluorescence is measured. The target specific protein thiol redox state can then be ratiometrically calculated as F‐MAL/F‐NHS. Alternatively, the RedoxiFluor assay uses two thiol‐reactive fluorescent reporters to measure target‐specific protein thiol redox state in percentages and moles (Tuncay et al., 2022), providing enhanced interpretation compared to fold changes provided by the ALISA. Reduced thiols are initially labelled with a fluorescent maleimide (F‐MAL1) reporter and reversibly oxidised thiols are then reduced and labelled with a spectrally distinct fluorescent maleimide reporter (F‐MAL2). A capture‐antibody‐functionalised solid support is used to bind the target protein thiol from a biological sample. The thiol redox‐state‐encoded F‐MAL reporters enable quantification of target‐specific protein thiol redox state as a percentage while a biotin‐conjugated detector antibody and recombinant protein standard curve enable calculation of target‐specific protein thiol redox state as a percentage and in moles. RedoxiFluor can also be used in array mode to quantify the redox state of several proteins as a percentage in a single assay. To date, these methods have been used to detect changes in target‐specific protein thiol oxidation in response to fertilisation in *Xenopus laevis* eggs (Noble et al., 2021) and lipopolysaccharide treatment in human monocytes (Tuncay et al., 2022). Future studies applying these methods to study redox signalling in skeletal muscle will be of interest.

## 4 APPROACHES TO STUDYING THE ROLE OF REDOX PTMS

Beyond identifying redox modifications, causal validation of cysteine redox modifications is critical to understanding their role in biology. Redox‐dead transgenic mouse models in which critical cysteine residues are replaced by amino acids, usually serine or alanine, so they can no longer undergo reversible oxidation provide an approach to studying the biological role of protein cysteine oxidation in vivo. For example, loss of protein kinase A type I regulatory subunit α (PKARIα) oxidation in redox‐dead Cys17Ser PKARIα knock‐in mice impairs blood vessel re‐growth after hindlimb ischaemia and reduces tumour growth and vascularization compared with wild‐type mice (Burgoyne, Rudyk et al., 2015), highlighting a role for PKARIα oxidation in ROS‐mediated angiogenesis.

While the generation of redox‐dead transgenic mice provides a mechanism to study the biological roles of protein cysteine oxidation, a limitation of this approach is that cysteine substitution also disrupts non‐redox cysteine roles (i.e., structural disulfide or metal binding). Emerging chemical biology strategies that selectively induce or block specific cysteine redox modifications offer the potential to study the role of site‐specific modifications in cellular processes without perturbing other non‐redox processes (Yang, 2025). Bioorthogonal cleavage chemistry, combined with genetic code expansion, has emerged as a transformative tool for precise protein manipulation in living systems (Wang et al., 2021). This approach enables incorporation of unnatural amino acids into proteins at defined sites and controlled activation of proteins by releasing functional groups on demand (Wang et al., 2019) and could be leveraged to achieve site‐specific incorporation of sulfenic acid (SOH) modifications within a protein of interest, presenting a gain‐of‐function model to study the role of protein cysteine modification. By replacing a target cysteine residue with chemically ‘caged’ unnatural amino acids, the redox‐sensitive SOH modification could be masked during protein synthesis and later activated via bioorthogonal cleavage chemistry under controlled conditions (Yang, 2025). Alternatively, the development of redox‐targeted covalent inhibitors provides a means to selectively capture and block SOH‐modified proteins, thereby preventing downstream redox‐dependent signalling steps (Yang, 2025). However, existing tools are primarily designed for broad profiling of SOH across the proteome, rather than selective targeting. Together, these strategies may facilitate causal validation of SOH‐mediated redox signalling, providing mechanistic insight into the roles of cysteine oxidation in redox regulation in biological processes.

## 5 CONCLUSION AND FUTURE DIRECTIONS

Redox proteomic approaches have enabled detailed characterisation of protein cysteine oxidation patterns in skeletal muscle, providing valuable insight into the molecular mechanisms underlying muscle function, adaptation and pathology. Future studies that combine redox proteomics with techniques for site‐specific manipulation of cysteine residues will allow for more direct investigation of how individual redox modifications influence protein function. By linking changes in redox state with functional outcomes, these approaches will help to delineate the precise role of cysteine oxidation in muscle physiology and may reveal key pathways altered in pathological conditions, opening new avenues for targeted therapeutic intervention. Integrating redox proteomics with other omics technologies, including phosphoproteomics and transcriptomics, will further enhance our understanding of skeletal muscle redox regulatory networks. Mapping the links between cysteine oxidation and other PTMs will enable the identification of broader regulatory networks that coordinate muscle function in both health and disease. Given the central role of redox signalling in regulating muscle function, targeting cysteine oxidation represents a promising therapeutic strategy for diseases linked to impaired redox regulation (e.g., muscular dystrophy). Future research should explore redox‐based interventions, including antioxidants or compounds that selectively modulate cysteine oxidation, to prevent or treat muscle‐related diseases. The development of small molecules that target redox‐sensitive cysteine residues or restore redox homeostasis could provide novel therapeutic options. Finally, because the functional consequences of cysteine oxidation are often difficult to predict due to the complexity of redox modifications and their effects on protein conformation, advanced computational approaches, such as molecular dynamics simulations and structural bioinformatics, offer powerful tools to model these changes. Given that contemporary redox proteomic approaches provide large amounts of data, these approaches will be important for indicating functional effects for further study. Leveraging these technologies will allow researchers to predict how specific cysteine oxidation events influence the structural and functional dynamics of key skeletal muscle proteins, providing mechanistic insight into redox regulation in muscle physiology.

## AUTHOR CONTRIBUTIONS

Sophie C. Broome wrote the first draft of the manuscript and prepared the figures. Elizabeth G. Reisman, Malcolm J. Jackson and John A. Hawley reviewed the manuscript and provided feedback. Elizabeth G. Reisman also contributed to figure preparation. All authors reviewed the final version of the manuscript. All authors have read and approved the final version of this manuscript and agree to be accountable for all aspects of the work in ensuring that questions related to the accuracy or integrity of any part of the work are appropriately investigated and resolved. All persons designated as authors qualify for authorship, and all those who qualify for authorship are listed.

## CONFLICT OF INTEREST

None declared.

## FUNDING INFORMATION

No funding was received for this work.

## GENERATIVE AI STATEMENT

Generative AI was used during the preparation of this manuscript to assist with grammar, spelling and language refinement.
